# Quantitative investigation of the affinity of human respiratory syncytial virus phosphoprotein C-terminus binding to nucleocapsid protein

**DOI:** 10.1186/s12985-014-0191-2

**Published:** 2014-11-19

**Authors:** Adam B Shapiro, Ning Gao, Nichole O’Connell, Jun Hu, Jason Thresher, Rong-Fang Gu, Ross Overman, Ian M Hardern, Graham G Sproat

**Affiliations:** Biology Department, Infection Innovative Medicines Unit, AstraZeneca R&D Boston, Waltham, MA USA; Reagents & Assay Development, Discovery Sciences, AstraZeneca R&D Boston, Waltham, MA USA; Structure & Biophysics, Discovery Sciences, AstraZeneca R&D Boston, Waltham, MA USA; Reagents & Assay Development, Discovery Sciences, AstraZeneca Pharmaceuticals, Macclesfield, Cheshire UK

**Keywords:** Respiratory syncytial virus, Nucleocapsid, Phosphoprotein, Phosphorylation, Protein-protein interaction, Fluorescence anisotropy, Surface plasmon resonance, Nuclear magnetic resonance spectroscopy

## Abstract

**Background:**

There are no approved small molecule drug therapies for human respiratory syncytial virus (hRSV), a cause of morbidity and mortality in at-risk newborns, the immunocompromised, and the elderly. We have investigated as a potential novel hRSV drug target the protein-protein interaction between the C-terminus of the viral phosphoprotein (P) and the viral nucleocapsid protein (N), components of the ribonucleoprotein complex that contains, replicates, and transcribes the viral RNA genome. Earlier work by others established that the 9 C-terminal residues of P are necessary and sufficient for binding to N.

**Methods:**

We used a fluorescence anisotropy assay, surface plasmon resonance and 2-D NMR to quantify the affinities of peptides based on the C terminus of P for RNA-free, monomeric N-terminal-truncated N(13-391). We calculated the contributions to the free energies of binding of P to N(13-391) attributable to the C-terminal 11 residues, phosphorylation of the C-terminal 2 serine residues, the C-terminal Asp-Phe, and the phenyl ring of the C-terminal Phe.

**Results:**

Binding studies confirmed the crucial role of the phosphorylated C-terminal peptide D(pS)DNDL(pS)LEDF for binding of P to RNA-free, monomeric N(13-391), contributing over 90% of the binding free energy at low ionic strength. The phenyl ring of the C-terminal Phe residue contributed an estimated -2.7 kcal/mole of the free energy of binding, the C-terminal Asp-Phe residues contributed -3.8 kcal/mole, the sequence DSDNDLSLE contributed -3.1 kcal/mole, and phosphorylation of the 2 Ser residues contributed -1.8 kcal/mole. Due to the high negative charge of the C-terminal peptide, the affinity of the P C-terminus for N(13-391) decreased as the ionic strength increased.

**Conclusions:**

The results support the idea that the interaction of the C-terminal residues of P with N constitutes a protein-protein interaction hotspot that may be a suitable target for small-molecule drugs that inhibit viral genome replication and transcription.

## Background

Human respiratory syncytial virus (hRSV) is a non-segmented negative sense RNA virus in the order *Mononegavirales*, the family *Paramyxoviridae* and the genus *Pneumovirus*, and is the cause of severe respiratory tract infections among newborns, immunocompromised people, and the elderly [1]. Current treatments are limited to antibody prophylaxis with palivizumab for high-risk newborns, general antiviral therapy with ribavirin, and supportive care. Efforts to develop anti-RSV vaccines and new drugs to prevent and treat the disease have so far been unsuccessful [2–4].

The genome of hRSV codes for 11 proteins. Four of these, the polymerase (L), nucleocapsid protein (N), phosphoprotein (P) and transcription anti-termination factor (M2-1), along with the RNA genome, make up the ribonucleoprotein (RNP) complex responsible for transcription and replication of the RNA. Several types of low molecular weight compounds have been identified that appear to target the RNP. Mutations conferring resistance to the inhibitory effect of YM-53404 and related compounds on viral replication were found in the polymerase L [5,6]. Mutations conferring resistance to RSV604 are found in the nucleocapsid N protein [7]. Liuzzi et al. [8] identified inhibitors of the mRNA capping function of the L protein, resistance mutations to which are found in the L protein. Thus the RNP is a viable target for compounds that inhibit RSV replication.

Several protein-protein interactions are involved in assembly of RNP. The N protein forms helical oligomers around which the RNA is wrapped [9,10]. P protein oligomerizes [11,12] and interacts with N [12–15], M2-1[16–20], and L [21–23]. M2-1 is a tetramer [19,24] and interacts with N [25–27] as well as P. These protein-protein interactions may offer suitable targets for antiviral drug discovery.

Protein-protein interactions can be difficult to disrupt with low molecular weight drugs if the interactions occur over a large, flat surface. Protein-protein interaction hotspots, confined areas with deep topological features, however, offer greater potential for drug discovery [28]. The information available concerning the interaction of the C-terminus of the RSV P protein with the N protein suggests that this interaction may occur at such a hotspot. A putative P binding region on the N-terminal core domain of N was identified by Galloux et al. [13], including Lys 46, Met 50, Ile 53, Ser 131, Arg 132, Tyr 135, Arg 150, and His 151. Mutations of several residues in this domain to alanine reduced both viral RNA synthesis and the N-P interaction *in vivo* and *in vitro*. On the crystal structure of N, these residues are clustered together, forming a potential P binding site with a hydrophobic cavity surrounded by several basic side chains. Remarkably, only the C-terminal 9 residues of P (Asp 233 to Phe 241) are necessary for binding to N in pull-down assays [13,15]. Moreover, changing just the C-terminal phenylalanine residue of P to alanine abrogates both N binding and viral RNA synthesis [13]. These observations strongly support the existence of a P-N binding hotspot involving the C-terminus of P and the aforementioned N-terminal core domain of N.

The C-terminal 11 residues of P (Asp-Ser-Asp-Asn-Asp-Leu-Ser-Leu-Glu-Asp-Phe) include 2 serine residues, Ser 232 and Ser 237, both of which have been observed to be phosphorylated, although the importance of their phosphorylation is unclear. Mazumder et al. [29] showed that purified recombinant P was selectively phosphorylated on Ser 237 by casein kinase II. Mazumder and Barik [30] found that mutation of Ser 237 to Ala abrogated RNA transcription by reconstituted RNP *in vitro*, whereas mutation of Ser 232 to Ala had no effect. In contrast, Barik et al. [31] found that Ser 232 was the major site of P phosphorylation when it was expressed in human cells or when unphosphorylated recombinant P was treated with a crude human cell extract. Sánchez-Seco et al. [32] found that Ser 232, not Ser 237, was the main site of phosphorylation of recombinant P *in vitro* by casein kinase II. Villanueva et al. [33] found that phosphorylation of P expressed in human cells was mainly on Ser 232 and to a lesser extent on Ser 237, based on the decreased level of phosphorylation when either residue was changed to Ala, but that mutation of either of these residues had little effect on viral transcription or replication. Only mutation of both residues combined with mutation of other probable sites of serine phosphorylation elsewhere in the protein substantially reduced, but did not completely eliminate, transcription and replication. Finally, Lu et al. [34] found that eliminating phosphorylation of Ser 232 and Ser 237 by mutation reduced P phosphorylation by 80% in infected cells, but had only modest effects on reporter gene expression and the N-P interaction. The mutant virus showed impaired replication in rodents compared with the wild-type virus, however. The inconsistencies in the results reported in the literature on this topic do not allow us to draw definitive conclusions regarding either the extent of phosphorylation of Ser 232 and Ser 237 or the roles of these residues in the functions of P, including its interaction with N.

We have taken a quantitative biophysical approach to investigate the P-N protein-protein interaction with regard to the effect of phosphorylation of the C-terminal N-binding peptide of P on residues 232 and 237 and the importance of the C-terminal Phe residue. In the process, we demonstrate 3 practical measurement systems – fluorescence anisotropy, surface plasmon resonance, and 2-D NMR - that can be used as the first steps in drug discovery programs to identify inhibitors of the interaction.

## Results

### Fluorescence anisotropy measurements of P C-terminal peptide binding to N(13-391)

**Figure 1:**
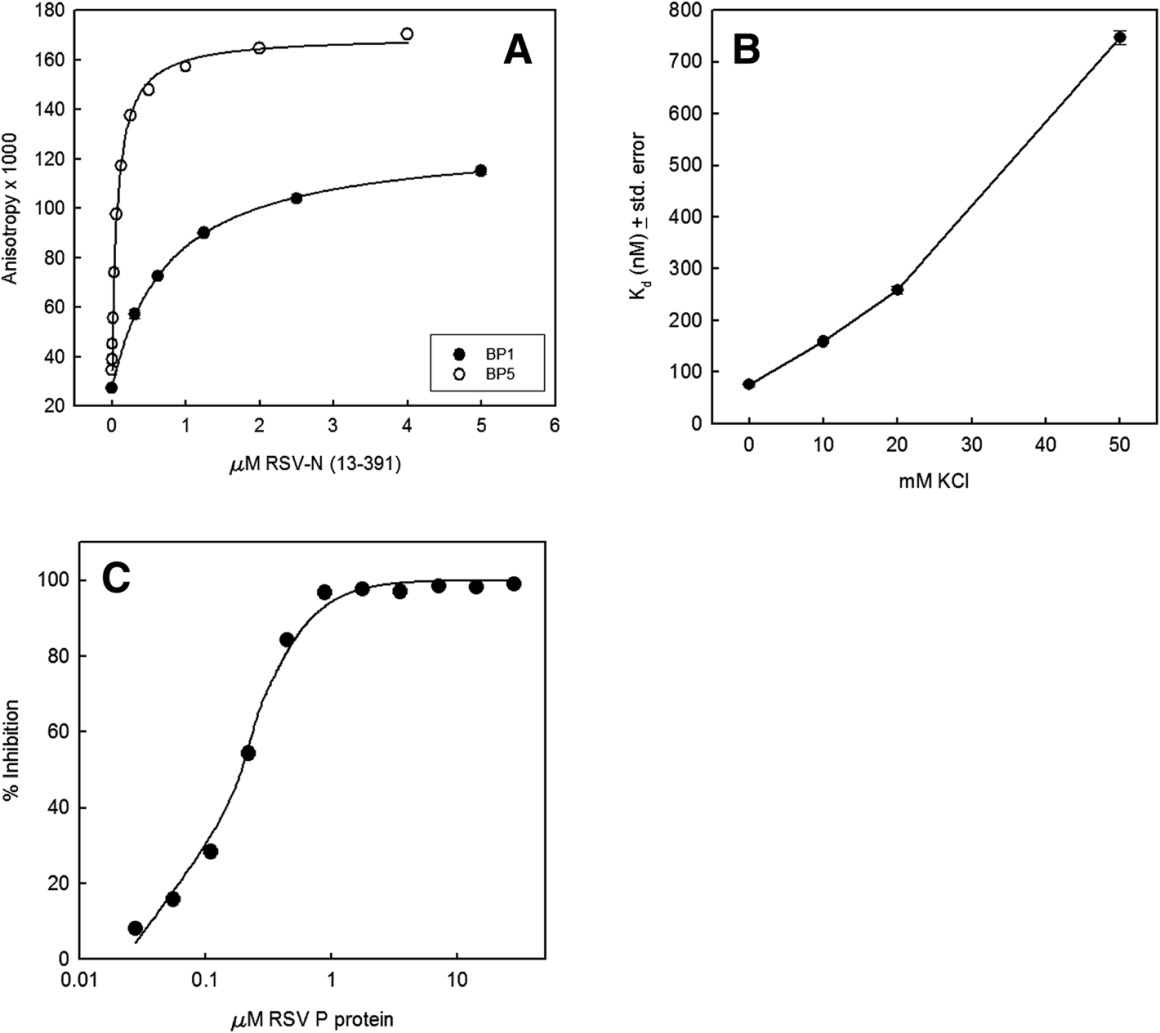
**Fluorescence anisotropy measurements of P C-terminal peptide binding to N(13-391), effect of KCl, and competition by full-length P. (A)** Affinity of unphosphorylated and doubly phosphorylated P-derived C-terminal peptides BP1 and BP5, respectively, for N(13-391). Data points and error bars represent the means and standard deviations, respectively, of 3 replicates. The error bars are mostly hidden by the data points. The K_d_s for BP1 and BP5 were 760 ± 20 nM and 78 ± 5 nM, respectively (best-fit value ± standard error of fit). **(B)** Effect of KCl concentration on affinity of BP5 for N(13-391). K_d_s were measured with N(13-391) concentrations ranging from 1.95 to 2000 nM in triplicate. The data were fit as above. Data points and error bars represent the best-fit K_d_s and the standard errors of the fits, respectively. **(C)** Inhibition of BP5 binding to N(13-391) by full-length P protein. The IC_50_ was 180 ± 10 nM (best-fit value ± standard error of fit).

Peptides having the sequence of the C-terminal 11 residues of RSV P protein (DSDNDLSLEDF) were labeled on the N-terminus with the fluorophore BODIPY FL for fluorescence anisotropy-based measurements of binding to N(13-391), an N-terminally truncated construct of RSV N protein that does not oligomerize [35]. The truncation makes the protein easier to overexpress in soluble form in bacteria than full-length N. When purified from bacteria, the truncated N proteins used in this study had no detectable RNA associated with them. One peptide was unphosphorylated (BP1). The other was phosphorylated on both serine residues (BP5), corresponding to Ser 232 and Ser 237 of the P protein. Both peptides bound to the N(13-391) construct (Figure 1A). The affinity of the doubly phosphorylated peptide BP5 (K_d_ = 78 ± 5 nM) was about 10 times that of the unphosphorylated peptide BP1 (K_d_ = 760 ± 20 nM). This result indicates that phosphorylation of the serine residues in the C-terminus of P is likely to affect the affinity of the N-P binding interaction. The anisotropy change was also larger for BP5 than BP1. Subsequent experiments were performed with BP5 because of the lower N(13-391) concentration required to perform competition experiments and larger signal compared with BP1.

Considering the highly negative charge of the doubly phosphorylated P C-terminal peptide due to the presence of 2 phosphate groups and 5 acidic side chains within the 11 residue peptide, we reasoned that electrostatic interactions with N would likely play a significant role in the interaction between them. Moreover, the putative P binding site on N contains several basic amino acid side chains [13]. In support of this hypothesis, we found that the affinity of BP5 for N(13-391) diminished as the ionic strength increased (Figure 1B). Subsequent experiments were performed in a low-ionic strength buffer in order to maximize the affinity of BP5 for N(13-391), thereby minimizing the amount of protein required.

### Effect of P C-terminal peptide phosphorylation on binding to N(13-391) measured by fluorescence anisotropy

The effect of phosphorylation of each of the two serine residues in the C-terminal peptide on its affinity for N(13-391) was investigated by competition with BP5 binding (Table 1). Phosphorylation of either serine residue substantially increased the affinity of the peptide compared with the unphosphorylated peptide, 7-fold for the N-terminal serine and 10-fold for the C-terminal serine. Phosphorylation of both serine residues resulted in an additional affinity enhancement of approximately 3-fold greater than observed for phosphorylation of one residue. As a result, the affinity of the doubly phosphorylated peptide was 20-25-fold higher than the affinity of the unphosphorylated peptide. This is consistent with the above observation that the affinity of doubly phosphorylated BP5 is 10-fold higher than the affinity of unphosphorylated BP1.

**Table 1:** **Competition by RSV-P C-terminal-derived peptides with BP5 binding to RSV-N(13-391)**

	**IC**_**50**_ **(μM) ± standard error**	
**Peptide sequence**	**Fluorescence anisotropy**	**SPR**
DSDNDLSLEDF	15.3 ± 0.4	230 ± 30
DSDNDLSLEDA	>200	ND
D(pS)DNDLSLEDF	2.17 ± 0.04	44 ± 3
DSDNDL(pS)LEDF	1.51 ± 0.03	31 ± 2
D(pS)DNDL(pS)LEDF	0.61 ± 0.01, 0.74 ± 0.02	13.6 ± 0.3
D(pS)DNDL(pS)LEDA	69 ± 2	ND
DF	1450 ± 50	ND

Competition was measured by the fluorescence anisotropy assay and with N(13-391) binding to P by SPR. Competitor peptide concentrations ranged from 0.2 to 200 μM. The IC_50_ for D(pS)DNDL(pS)LEDF was measured twice using fluorescence anisotropy. IC_50_s from the fluorescence anisotropy assay are approximately twice the K_d_s of the competitors because the N(13-391) concentration was set at the K_d_ for the interaction of N(13-391) with BP5. ND, not done.

The K_d_s of the peptides in the fluorescence anisotropy experiments are approximately ½ the IC_50_s because the N(13-391) concentration used was equal to its K_d_ with BP5. Therefore, the K_d_s of DSDNDLSLEDF and D(pS)DNDL(pS)LEDF were about 7.6 and 0.34 μM, respectively. These K_d_s are considerably higher than the K_d_s of the same peptides bearing an N-terminal BODIPY FL fluorophore, BP1 (0.76 μM) and BP5 (0.078 μM), respectively. This is most likely due to the hydrophobic character of the fluorophore, which tends to enhance the affinity of a ligand as long as the fluorophore does not cause a steric clash.

### Effect of changing the P C-terminal peptide’s C-terminal residue on binding to N(13-391) measured by fluorescence anisotropy

The C-terminal phenylalanine residue of P is crucial for binding of P to N, and mutation to alanine abrogates binding and viral replication [13]. The effect of changing the C-terminal Phe residue to Ala on competition by the P C-terminal peptide with BP5 binding to N(13-391) was substantial (Table 1). For unphosphorylated peptides, the IC_50_ increased by more than 13-fold when Phe was changed to Ala. The IC_50_ of the Ala-modified peptide exceeded the highest concentration tested (200 μM). Inhibition by 200 μM peptide was about 25%, so the true IC_50_ can be estimated to have been approximately 600 μM, assuming a Hill slope of 1, which was typical for these measurements. Thus the IC_50_ shifted upward about 40-fold when Phe was replaced with Ala in the unphosphorylated peptide. For the phosphorylated peptides, changing Phe to Ala resulted in an upward shift of the IC_50_ by approximately 100-fold. These results are consistent with the literature showing that the C-terminal Phe of P is a crucial binding determinant for its interaction with N.

### Competition by full-length P protein with P C-terminal peptide binding measured by fluorescence anisotropy

Full-length P protein produced in insect cells was tested for its ability to compete with BP5 binding to N(13-391). As determined by mass spectrometry, the P protein consists of a mixture of unphosphorylated, singly and doubly phosphorylated species, with the singly phosphorylated species most abundant (data not shown). The locations of the phosphorylated residues are unknown and may differ between molecules. Figure 1C shows that this P protein competed with an IC_50_ of 180 nM (K_d_ ~90 nM). Interestingly, this is only about 4-fold greater affinity than was measured for the doubly phosphorylated C-terminal peptide D(pS)DNDL(pS)LEDF. This observation is consistent with the hypothesis that the interaction of P with N is largely, if not entirely, determined by the last several residues at the C-terminus of P.

### Surface plasmon resonance measurements

**Figure 2:**
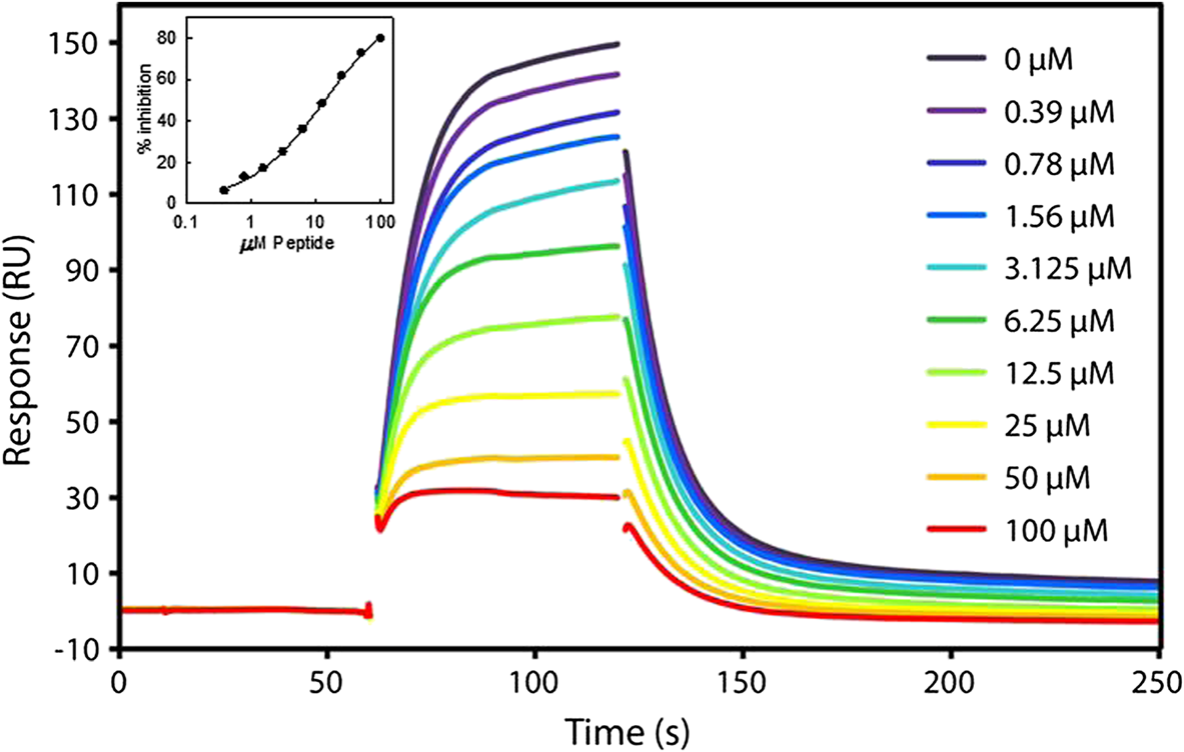
**Inhibition of N(13-391) binding to immobilized P protein by D(pS)DNDL(pS)LEDF peptide measured by SPR.** Concentrations of the peptide are indicated on the figure. The IC_50_ (curve shown in inset) was 13.7 ± 0.3 μM (best-fit value ± standard error of the best-fit value). The concentration of N(13-391) was 100 nM. Injection spikes have been deleted from the image for clarity.

The P C-terminus-derived peptides were tested for their ability to compete with soluble N(13-391) binding to immobilized P using SPR. The SPR results are consistent, in terms of rank order of potencies, with the results from the fluorescence anisotropy measurements, although the IC_50_s are higher for the SPR measurements (Figure 2 and Table 1). This is likely due to the higher ionic strength used in the SPR experiments (see Figure 1B). The SPR results demonstrate that the fluorescence anisotropy assay of the inhibition by peptides of BP5 binding to N(13-391) is a reliable surrogate for the inhibition by peptides of N(13-391) binding to P.

### 2-D NMR measurements

**Figure 3:**
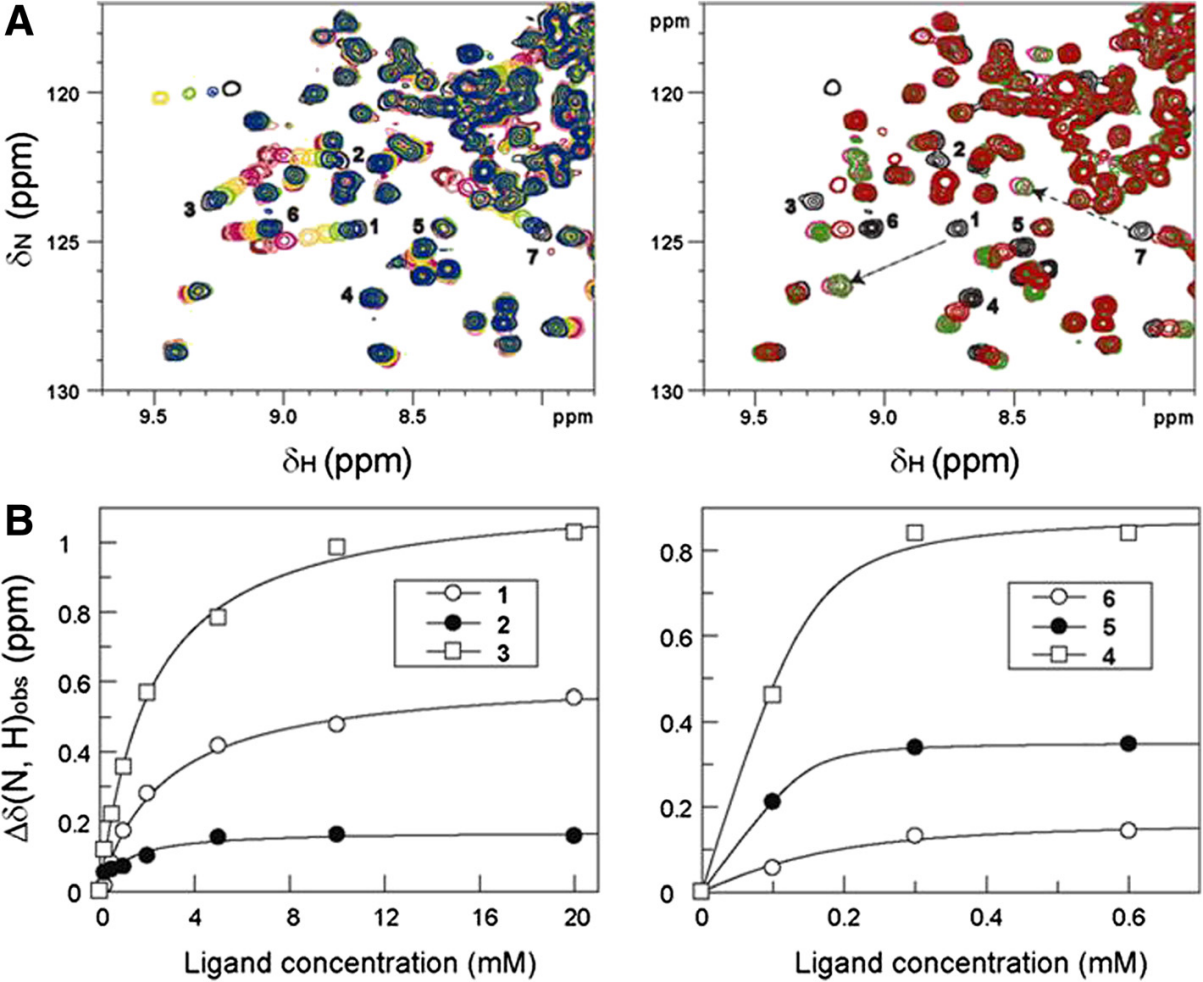
**Binding of Asp-Phe (left) and D(pS)DNDL(pS)LEDF (right) to N(31-252). (A)** Contour plots of backbone amide resonances of 0.15 mM ^15^N-labeled N(31-252) obtained from two-dimensional [^15^N, ^1^H] TROSY spectra recorded at a range of ligand concentrations. Numbered resonances correspond to the graphs in panel **B**. Dotted arrows denote the putative directions of shift for resonances 1 and 7. The missing resonances of 1 and 7 in the presence of 0.1 mM D(pS)DNDL(pS)LEDF indicate the binding interaction is in the intermediate exchange regime [36]. **(B)** Chemical shift perturbations Δδ(N, H)_obs_ of selected amide resonances of ^15^N-labeled N(31-252) as a function of ligand concentration. The symbols represent the experimental data and the lines are global best-fit theoretical curves for a simple binding isotherm. The best-fit values ± standard error of the fit of K_d_s were 2.6 ± 0.3 mM for Asp-Phe (left) and 74 ± 4 μM for D(pS)DNDL(pS)LEDF (right).

**Figure 4:**
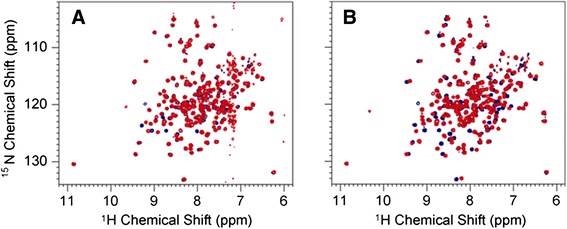
**Backbone amide resonances of** ^**15**^ **N-labeled N(31-252) from two-dimensional [** ^**15**^ **N,** ^**1**^ **H] TROSY spectra.** Blue, no ligand. Red, peptide ligand. The concentration of N(31-252) was 0.15 mM. **(A)** 2 mM Asp-Phe. **(B)** 0.3 mM D(pS)DNDL(pS)LEDF.

The importance of the C-terminal Phe residue for N binding was studied by measuring the ability of the P-derived C-terminal dipeptide Asp-Phe to compete with BP5 binding to N(13-391). The IC_50_ was 1.45 mM (Table 1). Two-dimensional NMR experiments with ^15^N-labeled N(31-252) confirmed that this dipeptide binds to N(31-252), as shown by the shift in position of several crosspeaks (Figure 3A). When the longer peptide D(pS)DNDL(pS)LEDF was used, additional crosspeaks shifted position (Figure 4). This result demonstrates that the longer peptide makes more contacts with the P binding site of N(31-252) than the dipeptide. The K_d_s of Asp-Phe and D(pS)DNDL(pS)LEDF measured by NMR were 2.6 mM and 74 μM, respectively (Figure 3B). These K_d_s were higher than those measured by the fluorescence anisotropy assay because of the higher ionic strength of the buffer in which the NMR experiments were performed. As expected, this ionic strength effect was greater for the more highly charged peptide D(pS)DNDL(pS)LEDF than for Asp-Phe.

## Discussion

Using the relationships ΔG = RT ln K_d_ and IC_50_ = 2 × K_d_, we can obtain approximate values for ΔG of binding for the components of the C-terminal peptide of P to RNA-free N(13-391) in 10 mM Tris-HCl (pH 7.5) at 21°C. This calculation shows that Asp-Phe (DF) contributes -3.8 kcal/mole out of -8.7 kcal/mole for D(pS)DNDL(pS)LEDF. The contribution of the phenyl ring of the Phe residue can be estimated by comparing ΔG of binding for D(pS)DNDL(pS)LEDF (-8.7 kcal/mole) to that for D(pS)DNDL(pS)LEDA (-6.0 kcal/mole), hence ΔΔG = -2.7 kcal/mole, corresponding to a K_d_ for the phenyl ring of 10 mM. The ΔG of binding attributable to DSDNDLSLE can be estimated by subtracting the ΔG of binding of DF (-3.8 kcal/mole) from the ΔG of binding of DSDNDLSLEDF (-6.9 kcal/mole) to obtain a value of -3.1 kcal/mole for this part of the C-terminal peptide, which contains 5 acidic side chains (not counting the C-terminus) that could interact with basic residues in the P binding site of N(13-391). The ΔG of binding contributed by phosphorylation of both Ser residues can be estimated by comparing ΔG of binding for DSDNDLSLEDF (-6.9 kcal/mole) to that for D(pS)DNDL(pS)LEDF (-8.7 kcal/mole), hence ΔΔG = -1.8 kcal/mole. Interestingly, these estimates indicate that the phenyl ring of the C-terminal Phe residue contributes more free energy of binding than both phosphate moieties, that over 40% of the free energy of binding of D(pS)DNDL(pS)LEDF comes from the DF dipeptide, and that over 90% of the free energy of binding of the full-length P used in this experiment can be attributed to the D(pS)DNDL(pS)LEDF peptide. These calculations are oversimplified by not taking all factors into account, such as the fact that DF and D(pS)DNDL(pS)LEDF have free N-termini that are parts of amide bonds in the longer peptides and the full-length protein, respectively, and the unknown phosphorylation status of the C-terminal serine residues of the full-length P. Nevertheless, the calculations are helpful in demonstrating that most of the affinity of P for N(13-391) is due to the last several residues at the C-terminus of P, and that much of the affinity of these C-terminal residues is due to the binding of the last 2 residues. This observation is consistent with the idea that the N-P interaction can be considered to be a protein-protein interaction hotspot.

The relatively small energetic contribution of Ser 232 and Ser 237 phosphorylation to the affinity of D(pS)DNDL(pS)LEDF for binding to N(13-391) (-1.8 kcal/mole), combined with the observation that phosphorylation of either of the serine residues has nearly as much effect on the affinity as phosphorylation of both, suggests a possible explanation for the inconsistent results reported in the literature on the importance of Ser 232 and Ser 237 for RSV transcription and replication (see Introduction). The extent to which P C-terminal phosphorylation affects RNP assembly will depend on the local concentrations of the P and/or N proteins. If at least one of these concentrations is well in excess of the N-P binding K_d_ when P is unphosphorylated, then the interaction will not be significantly affected by phosphorylation. In contrast, if the concentrations of both of the proteins are lower than the K_d_, then phosphorylation, by lowering the K_d_, will drive the binding interaction. It may be that the N and P concentrations present in the various reported experiments differed sufficiently for the extent of P C-terminal phosphorylation to affect the efficiency of RNP assembly differently between them. For example, Mazumder and Barik [30] used *in vitro* reconstituted RNP, whereas Lu et al. [34] used infected cells.

In a comparison of the sequences of residues 231-241 in 163 publicly available sequences of RSV P protein in PubMed, the 3rd-to-last residue, Glu 239, is frequently conservatively replaced by Asp, and there is one instance each of Asn 234 being replaced by Asp and Ser 237 being replaced by Leu. The other residues are invariant. Similarly, the residues of N protein identified by Galloux et al. [13] as being involved in binding to P - Lys 46, Met 50, Ile 53, Ser 131, Arg 132, Tyr 135, Arg 150, and His 151 – are completely conserved among 126 published full-length N sequences in PubMed. This high degree of sequence conservation is consistent with the importance of P residues 231-241 and the aforementioned N residues for binding of P and N.

## Conclusion

Three biophysical methods were used in this research: (1) a fluorescence anisotropy-based binding assay to measure competition by P-derived peptides and full-length P with binding of BP5 (D(pS)DNDL(pS)LEDF labeled on the N-terminus with BODIPY FL) to N(13-391), (2) surface plasmon resonance (SPR) to measure the competition by P-derived peptides with binding of N(13-391) to P, and (3) 2-D NMR to measure direct binding of Asp-Phe D(pS)DNDL(pS)LEDF to N(31-252). Each of these methods is suitable for screening compounds for binding to the N-P protein-protein interaction hotspot. Because of their relatively low throughput, SPR and NMR are most useful for screening small libraries of hundreds of very low molecular weight (<300 Da) fragments. The fluorescence anisotropy assay can be used to screen much larger libraries consisting of hundreds of thousands of compounds because it is a high-throughput assay. The active samples from such a screen could then be counterscreened to remove false positives due to detection artifacts using SPR and/or NMR. Thus the methods used here could be used to begin a drug discovery program aimed at identifying competitive inhibitors of the binding of RSV P protein to N that work by occupying the P binding site of N.

## Methods

### Peptides

Oligopeptides corresponding to residues 231-241 of RSV P protein, including phosphorylation, were synthesized and purified by Biopeptide Co. Inc. (San Diego, CA). Purities of peptides were at least 95%. N- and C-termini were uncapped. Asp-Phe was from Sigma.

### Preparation of BODIPY-FL-labeled peptides 1 and 5 (BP1 and BP5)

One μmole (1.3 mg) of peptide 1 (DSDNDLSLEDF) and 10 μmoles (5 mg) of BODIPY FL sulfosuccinimidyl ester (SSE) (Life Technologies) were dissolved in 100 μl of fresh 0.1 M NaHCO_3_ and reacted for 1 hour in darkness at room temperature. Peptide 5 (D(pS)DNDL(pS)LEDF), where (pS) is phosphoserine, (1.2 mg, 0.84 μmole) and 10 μmoles of BODIPY FL SSE were dissolved in 400 μl of fresh 0.2 M NaHCO_3_ + 50 μl of dimethyl sulfoxide and reacted for 2.75 hours at room temperature. In each case, the solution was applied to a 1 × 18-cm column of Sephadex LH-20 (Sigma) equilibrated with water. The column was eluted with water and fractions were collected. Liquid chromatography-mass spectrometry (LC-MS) was used to select fractions having the expected mass for the labeled peptide (1543 Da for BP1, 1701 Da for BP5) and little or no unlabeled peptide. For BP5, LC-MS also showed evidence of a 1682 Da species, suggesting loss of a fluorine atom, probably during the analysis. The concentration of the peptides was determined by the BODIPY FL absorbance at 504 nm using an extinction coefficient in water of 91,000 M^-1^cm^-1^.

### Mass spectrometry of peptides

The LC-MS analysis was performed on a Triple TOF 5600+ (AB Sciex, Redwood City, CA) equipped with a DuoSpray Ion Source and a Shimadzu LC 20-AD HPLC system (Shimadzu Scientific Instruments, Marlborough, MA). Separation was achieved on a 2.1 x 30 mm XBridge C18 column (Waters, Milford, MA) with a gradient of acetonitrile (5-90%) in 0.1% formic acid for 3 min following 1 min at 5% acetonitrile at a flow rate of 0.4 ml/min and column temperature of 30°C. LC-MS data was acquired in the TOF MS mode for m/z + from 100 to 2000. Nebulizer gas (GS1), heater gas (GS2), and curtain gas were set at 60, 70, and 30 psi, respectively. The source temperature was 600°C. Ion spray voltage was 5500 V. Declustering voltage was 100 V.

### Fluorescence anisotropy assays

Samples of 10 μl containing peptides and proteins as indicated were prepared in buffer composed of 10 mM Tris-HCl (pH 7.5) +0.01% Brij-35 detergent (Pierce Surfact-Amps-35, Thermo Fisher Scientific, Rockford, IL) in low-volume 384-well black polystyrene assay plates (Thermo Fisher Scientific, Hudson, NH). After a 5 minute incubation, fluorescence anisotropy was measured with a Pherastar plate reader (BMG Labtech, Cary, NC) equipped with a filter module having a 485 nm excitation filter and 520 nm emission filters with polarizers. The focal height was 10.8 mm. Each measurement used 50 flashes. The parallel and perpendicular detector gains were set to 392 and 366 in order to produce an anisotropy of about 0.03 in the absence of binding.

For measurements of fluorescent peptide K_d_s with RSV-N(13-391), BP1 and BP5 concentrations were 100 nM and 25 nM, respectively. The averages of triplicate fluorescence anisotropy measurements made at each RSV-N(13-391) concentration were fit to the equation

$$ \mathbf{y} = {\mathbf{y}}_o + \mathbf{a}\left[\mathbf{N}\right]/\left({\mathbf{K}}_{\mathbf{d}} + \left[\mathbf{N}\right]\right) $$

where **y** is the anisotropy, **y**_**o**_ is the anisotropy of peptide alone, **[N]** is the RSV-N(13-391) concentration, and **y**_**o**_ + **a** is the anisotropy of fully bound peptide. The K_d_s were not corrected for 18% and 14% decreases in the fluorescence intensities of BP1 and BP5, respectively, upon binding.

For IC_50_ measurements by competitors, the BP5 and RSV-N(13-391) concentrations were 25 and 75 nM, respectively. The averages of triplicate anisotropy measurements made at each competitor concentration were used to calculate % inhibition using the equation

$$ \%\kern0.62em \mathrm{inhibition} = 100\left[1\ \hbox{--}\ \left(\mathrm{r}\hbox{--} {\mathrm{r}}_{\min}\right)/\left({\mathrm{r}}_{\max}\hbox{--} {\mathrm{r}}_{\min}\right)\right] $$

where **r** is the anisotropy at the competitor concentration, **r**_**min**_ is the anisotropy with no RSV-N(13-391), and **r**_**max**_ is the anisotropy with no competititor. The % inhibition values were used to determine the IC_50_ by nonlinear regression using the equation

$$ \%\kern0.75em \mathrm{inhibition} = 100{\left[\mathrm{C}\right]}^{\mathrm{b}}/\left(\mathrm{I}{\mathrm{C}}_{50} + {\left[\mathrm{C}\right]}^{\mathrm{b}}\right) $$

where **[C]** is the competitor concentration and **b** is the Hill slope.

All fluorescence anisotropy measurements were performed at ambient temperature, approximately 21°C.

### Surface plasmon resonance (SPR)

SPR experiments were performed on a Biacore T200 using Series S NTA sensor chips (GE Healthcare). His_10_-RSV P protein was immobilized on the surface using the nickel-capture amine-couple approach. The immobilization buffer consisted of 10 mM HEPES-NaOH (pH 7.5), 150 mM NaCl, 0.1 mM EDTA, 1 mM tris(2-carboxyethyl)phosphine hydrochloride (TCEP) and 0.005% (v/v) Tween-20. The surface was activated for His-tag capture by injecting 500 μM NiSO_4_ for 60 s at a flow rate of 30 μl/min. Subsequent activation for amine coupling was achieved by injecting a mixture of 200 mM 1-ethyl-3-[3-dimethylaminopropyl]carbodiimide and 50 mM *N*-hydroxysuccinimide (NHS) for 7 min at flow rate 10 μl/min. His_10_-P protein was diluted in immobilization buffer to a final concentration of 6 μM. The protein was immobilized to ~6000 resonance units (RU) by applying short (1-4 μl) pulses of protein at 5 μl/min until the desired immobilization level was achieved. Residual NHS was blocked by a 7 min injection of 0.1 M Tris-HCl (pH 8.0) at 10 μl/min. Finally, the nickel was stripped from the surface using a 60 s injection of 350 mM EDTA at a flow rate of 30 μl/min.

Inhibition assays were carried out in 20 mM HEPES-NaOH (pH 7.5), 100 mM NaCl, 1 mM EDTA, 1 mM TCEP, 0.005% (v/v) Tween 20 and 1% (v/v) DMSO at a constant concentration of 100 nM RSV N(13-391). Peptides were mixed with N(13-391) in a 9-point concentration series of 2-fold dilutions from a top concentration of 100 μM. Three blanks were included for determining the maximum binding response. Samples were allowed to come to equilibrium for 30 min before injection at 40 μl/min for 60 s followed by a 180 s dissociation period. To monitor surface integrity, positive and negative controls consisting of 100 nM RSV N(31-252) and assay buffer, respectively, were run every 12 cycles. To correct for DMSO mismatches, three cycles of 8 solvent correction samples in the range 0-2% (v/v) DMSO were run at the beginning, middle and end of the experiment.

Data were analyzed using the Biacore T200 Evaluation Software V 2.0. Sensorgram data was reference-subtracted and solvent-corrected before the binding report point was extracted for each concentration at a time 4 s before the end of the injection. These data were used to calculate the percent inhibition according to the following equation: %Inhibition = 100*(1 – Response(conc)/Response_max), where Response_max is the average report point extracted from the 3 blank runs. Percent inhibition was plotted versus concentration and fit to a 4-parameter logistic Hill Equation (see above) for determining the peptide IC_50_.

### 2-D NMR

NMR spectra were acquired at 298 K with a 600 MHz NMR instrument (Bruker, Billerica MA) equipped with an AVANCE III console and a triple-resonance cryogenic probe. Samples were at pH 7.5 in a 25 mM HEPES buffer containing 100 mM NaCl, 1 mM dithiothreitol, and 1 mM EDTA. The concentration of ^15^N labeled RSV N(31-252) was 0.15 mM. Two-dimensional [^15^N, ^1^H] transverse relaxation optimized spectroscopy (TROSY) experiments were recorded with increasing ligand concentration [37]. Evolution times were approximately 53 ms in the ^1^H dimension and 29 ms in the ^15^N dimension. The total acquisition time was 28 min per experiment.

To estimate the dissociation constant (K_d_) from NMR experiments, ^15^N-RSV N(31-252) was titrated with a range of ligand concentrations. The weighted average of chemical shift changes (Δδ(N, H)_obs_) is calculated using the following equation:

$$ \varDelta \updelta {\left(\mathrm{N},\;\mathrm{H}\right)}_{\mathrm{obs}}=\sqrt{{\left(\varDelta {\updelta}_{\mathrm{HN}}\right)}^2+{\left(\varDelta {\updelta}_{\mathrm{N}}/5\right)}^2} $$

where Δδ_*HN*_ and Δδ_*N*_ are the chemical shift changes of amide protons and nitrogens, respectively. Then Δδ(N, H)_obs_ data were fitted by non-linear regression globally against the total concentration of the subtrate (L_T_) with the equation:

$$ \Delta \updelta {\left(\mathrm{N},\;\mathrm{H}\right)}_{\mathrm{obs}}=\Delta \updelta {\left(\mathrm{N},\;\mathrm{H}\right)}_{\max}\frac{\left({\mathrm{K}}_{\mathrm{d}}+{\mathrm{L}}_{\mathrm{T}}+{\mathrm{E}}_{\mathrm{T}}\right)\hbox{-} \sqrt{{\left({\mathrm{K}}_{\mathrm{d}}+{\mathrm{L}}_{\mathrm{T}}+{\mathrm{E}}_{\mathrm{T}}\right)}^2-4\left({\mathrm{L}}_{\mathrm{T}}{\mathrm{E}}_{\mathrm{T}}\right)}}{2{\mathrm{E}}_{\mathrm{T}}} $$

where Δδ(N, H)_max_ is the maximum chemical shift difference, E_T_ is the total concentration of N(31-352) in the solution, and L_T_ is the total concentration of ligand.

### DNA manipulations and plasmid construction

Plasmid DNA purification, PCR product purification, and gel extraction were performed using the QIAprep Spin Mini Plasmid Purification (Qiagen Inc., Valencia, CA), QuickStep™2 PCR Purification Kit (EdgeBio, Gaithersburg, MD), and Rapid Gel Extraction Kit (Marligen Biosciences, Ijamsville, MD), respectively. Restriction enzymes and rAPid Alkaline Phosphatase were from Roche Applied Science (Indianapolis, IN). Primers for PCR DNA were from Eurofins MWG Operon (Huntsville, AL). All PCRs were performed with High Fidelity PCR Master (Roche Applied Science) using reaction conditions specified by the manufacturer. All ligation reactions were performed using the Rapid DNA Ligation Kit (Roche Applied Science) according to the manufacturer’s instructions.

A DNA sequence codon optimized for expression in *E. coli*, encoding N-terminal His_6_-tagged RSV N(13-391) [strain A2, UniProtKB/Swiss-Prot: NCAP_HRSVA, P03418] was synthesized and cloned into pET-28b (GenScript, Piscataway, NJ). The plasmid was named pJT1128.

An expression plasmid was constructed for a N-terminal His_6_-tagged RSV N(31-252) as follows. A tobacco etch virus (TEV) protease cleavage recognition site was placed before the His_6_ tag for optional removal. This was accomplished by amplifying the gene encoding residues (31-252) by PCR from the codon-optimized sequence (see above for N(13-391)) using primers 31252For (5’-GGATT*CATATG*GATTCCCATCGACACCCCGAACTAC-3’) and 31253Rev (5’-GATGAT*GTCGAC*TTAGCCATACGCGTTCATGAACAGACCAGCAA-3’). The purified PCR product was digested with NdeI and SalI and ligated into a modified pET-28b plasmid to make plasmid pJT1324. The ligation mixture was transformed into *E. coli* DH5α chemically competent cells (Life Technologies, Grand Island, NY) and transformants were selected on Luria-Bertani (LB) agar supplemented with 30 μg/ml kanamycin. Transformants were analyzed by PCR, and plasmid DNA was isolated and sequenced for verification.

The gene sequence encoding a GST-tagged RSV P(1-241) construct was synthesized with codon optimization for expression in *E. coli* and cloned into pET-30a (BlueSky Bioservices, Worcester, MA). The plasmid was designated pJT1200.

The full length coding sequence (residues 1-241) for the human respiratory syncytial virus phosphoprotein P [strain A2, UniProtKB/Swiss-Prot: PHOSP_HRSVA, P03421] was synthesized *in vitro* by GeneArt AG (Life Technologies). The sequence was codon-optimized for *Spodoptera frugiperda* expression. The synthesized DNA fragment was restriction-cloned into a modified version of the pFastBac1 baculovirus expression vector (Life Technologies), downstream of an N-terminal His_10_ tag and a TEV protease cleavage recognition sequence.

### Co-expression of RSV N(13-391) and RSV P (1-241)

For protein production, the plasmids pJT1128 and pJT1200 were transformed into *E. coli* BL21(DE3)pLysS (EMD Chemicals, Gibbstown, NJ) and plated on LB medium containing 25 μg/ml kanamycin and 100 μg/ml ampicillin at 37°C overnight. A single colony of BL21(DE3)pLysS/pJT1128/pJT1200 was inoculated into a 100-ml culture of LB containing 25 μg/ml kanamycin and 100 μg/ml ampicillin and grown overnight at 37 °C. The overnight culture was diluted to OD_600_ = 0.1 in 4 × 1 L of LB containing 25 μg/ml kanamycin and 100 μg/ml ampicillin and grown at 30°C with aeration to mid-logarithmic phase (OD_600_ = 0.6). The culture was incubated on ice for 30 minutes and transferred to 18°C. IPTG was then added to a concentration of 0.5 mM. After overnight induction at 18°C, the cells were harvested by centrifugation at 5,000 *g* for 15 min at 25°C. Cell pastes were stored at –20°C.

### Purification of RSV N(13-391)

The frozen cell paste from 4 L of cell culture was suspended in 50 ml of Lysis Buffer consisting of 50 mM Tris-HCl (pH 8.0), 0.5 M NaCl, 5% (v/v) glycerol, and 1 EDTA-free protease inhibitor cocktail tablet (Roche Molecular Biochemical, Indianapolis IN). Cells were disrupted by French Press at 18,000 psi twice at 4°C. The crude extract was centrifuged at 150,000 *g* for 30 min at 4°C. The supernatant was applied at a flow rate of 2.0 ml/min onto a 5-ml HiTrap Ni^2+^ chelating column (GE Healthcare Life Sciences, Piscataway NJ) pre-equilibrated with Buffer A consisting of 50 mM Tris-HCl (pH 8.0), 0.5 M NaCl, and 5% glycerol. The column was then washed with Buffer A, and eluted with a linear gradient from 0 to 0.5 M imidazole in Buffer A. Fractions containing RSV N(13-391) were pooled and dialyzed against 1 L of Buffer B consisting of 50 mM Tris-HCl (pH 8.0), 1 mM EDTA, 1 mM dithiothreitol, and 5% glycerol. The dialyzed sample was loaded at a flow rate of 2.0 ml/min onto a 20-ml Q-Sepharose HP (HR16/10) column (GE Healthcare Life Sciences) pre-equilibrated with Buffer B. The column was washed with Buffer B and eluted with a linear gradient from 0 to 1 M NaCl in Buffer B. Fractions containing RSV N(13-391) were pooled and concentrated to 5 ml by Amicon® Ultracel-10K (Millipore, Billerica, MA). The 5 ml sample was applied at a flow rate of 1.5 ml/min to a 120-ml Superdex 200 (HR 16/60) column (GE Healthcare Life Sciences) pre-equilibrated and eluted with buffer consisting of 50 mM Tris-HCl (pH 8.0), 0.5 M NaCl, 1 mM EDTA, 1 mM dithiothreitol, and 5% glycerol. The fractions containing RSV N(13-391) were pooled and concentrated. The protein was characterized by SDS-PAGE and LC-MS, and the concentration was determined by the Bradford method [38]. The final yield of purified N(13-391) was 30 mg from 4 L of cell paste. The protein was stored at –80°C. GST-RSV P(1-241) was not detectably expressed in the cells and none copurified with RSV-N(13-391). The ratio of UV absorbance at 280 nm to absorbance at 260 nm (A280/A260) was 2.2. No RNA was detected in the N(13-391) protein when 2 μg of protein was run on an agarose gel and stained with ethidium bromide.

### Expression and purification of RSV N (31-252)

The method for overexpression of N (31-252) was the same as for coexpression of N(13-391) and P (1-241) (see above), except using only plasmid pJT1324 and omitting ampicillin. The frozen cell paste from 4 L of cell culture was suspended in 50 ml of Lysis Buffer consisting of 25 mM HEPES (pH 7.3), 0.5 M NaCl, 5% (v/v) glycerol, and 1 EDTA-free Protease inhibitor cocktail tablet. Cells were disrupted by French Press at 18,000 psi twice at 4°C. The crude extract was centrifuged at 150,000 *g* for 30 min at 4°C. The supernatant was applied at a flow rate of 2.0 ml/min onto a 5-ml HiTrap Ni^2+^ chelating column (GE Healthcare Life Sciences) pre-equilibrated with Buffer C consisting of 25 mM HEPES (pH 7.3), 0.5 M NaCl, and 5% glycerol. The column was washed with Buffer C, and eluted with a linear gradient from 0 M to 0.5 M imidazole in Buffer C. Fractions containing N (31-252) were pooled and concentrated to 5 ml. The 5-ml sample was applied at a flow rate of 1.5 ml/min to a 120-ml Superdex 200 (HR 16/60) column pre-equilibrated and eluted with buffer consisting of 25 mM HEPES (pH 7.3), 0.5 M NaCl, 1 mM EDTA, 1 mM dithiothreitol, and 5% glycerol. The fractions containing N (31-252) were pooled and concentrated. The protein was characterized by SDS-PAGE and LC-MS, and the concentration was determined by the Bradford method [38]. The final yield of purified N (31-252) was 135 mg from 4 L of cell paste. The protein was stored at –80°C. A280/A260 was 2.6. No RNA was detected in the N(31-252) protein when 2 μg of protein was run on an agarose gel and stained with ethidium bromide.

Uniformly ^15^N-labeled N (31-252) was prepared by growing cells in M9 minimal medium supplemented with 1 g/L of [^15^N] NH_4_Cl and 10 g/L glucose as the sole nitrogen and carbon sources. N (31-252) was purified as described above and stored in buffer consisting of 25 mM HEPES (pH 7.3), 0.1 M NaCl, 1 mM EDTA, and 1 mM dithiothreitol. The extent of isotope labeling was >93% ^15^N based on comparison of the observed mass with that expected from the sequence.

### Expression and purification of RSV P(1-241)

Routine *S. frugiperda* (Sf21) cell maintenance, generation of bacmid DNA, generation of recombinant baculoviruses, expression studies in 24-deep-well blocks and roller bottles were carried out essentially as described in [39], except that no gentamicin antibiotic was added to insect cell cultures. SF900II medium for Sf21 cells was from Invitrogen. Fetal bovine serum (FBS) was from PAA Laboratories.

Insect cell pellets from 2 L of culture were thawed from -80°C and resuspended by homogenization in 150 ml of Lysis Buffer consisting of 50 mM Tris-HCl (pH 8.0), 300 mM NaCl, 10 mM imidazole, 2 mM tris(2-carboxyethyl)phosphine (TCEP), 1 mM phenylmethylsulfonyl fluoride (PMSF), and 3 EDTA-free protease inhibitor tablets (Roche). Cells were lysed by sonication on ice (5 × 30-s bursts) and clarified by centrifugation at 50,000 *g* for 1h at 4°C. The supernatant was loaded at 0.2 ml/min onto a 2-ml column of Ni-NTA Superflow resin (Roche). The column was washed with lysis buffer followed by buffer D, consisting of 50 mM Tris-HCl (pH 8.0), 300 mM NaCl, 10 mM imidazole, and 2 mM TCEP, followed by additional washes at 20 mM and 40 mM imidazole in buffer D. Bound protein was eluted with 100% buffer E (buffer D with 300 mM imidazole). Fractions containing RSV P protein based on SDS-PAGE were pooled and further purified by applying at a flow rate of 0.5 ml/min to a 120-ml Superdex 75 (XK26/60) gel filtration column (GE Healthcare Life Sciences) pre-equilibrated and eluted with buffer F consisting of 50 mM Tris-HCl (pH 7.4), 200 mM NaCl, 15% glycerol, and 1 mM TCEP. The fractions with the highest purity as judged by SDS PAGE were pooled, snap-frozen in liquid nitrogen at 1.2 mg/ml, and stored at -80°C. The final yield of purified RSV P(1-241) was ~3 mg/L of cell paste. The His-tag was removed by TEV protease cleavage with a 1:200 ratio by weight of TurboTEV (Eton Bioscience, San Diego, CA) to RSV P(1-241) overnight at 4°C. The protease was removed by adsorption to immobilized Ni^2+^ resin.

For the BP5 competition assay, the P protein storage buffer was replaced by repeated concentration using centrifugal ultrafiltration with a Millipore Ultrafree 0.5 unit with Biomax-10 membrane (EMD Millipore, Billerica, MA) and dilution with 10 mM Tris-HCl (pH 7.5). The protein concentration was determined by the method of Bradford [38] using bovine serum albumin as the standard. Serial dilutions of P were prepared in 10 mM Tris-HCl (pH 7.5) +0.01% Brij-35.

## Abbreviations

P: Phosphoprotein
N: Nucleocapsid
N(13-391): Nucleocapsid with N-terminal truncation of 12 residues
N(31-252): Nucleocapsid construct consisting of residues 31-252
hRSV: Human respiratory syncytial virus
RNP: Ribonucleoprotein
BP1: BODIPY-FL labeled peptide 1 (DSDNDLSLEDF)
BP5: BODIPY-FL labeled peptide 5 (D(pS)DNDL(pS)LEDF)
SPR: Surface plasmon resonance
HPLC: High-performance liquid chromatography
TOF: Time-of-flight
2-D NMR: Two-dimensional nuclear magnetic resonance spectroscopy
LC-MS: Liquid chromatography-mass spectrometry
TCEP: Tris(2-carboxyethyl)phosphine hydrochloride
NHS: *N*-hydroxysuccinimide
RU: Resonance units
DMSO: Dimethyl sulfoxide
TROSY: Transverse relaxation optimized spectroscopy
TEV: Tobacco etch virus
LB: Luria-Bertani
GST: Glutathione-*S*-transferase
SDS-PAGE: Sodium dodecyl sulfate polyacrylamide gel electrophoresis
FBS: Fetal bovine serum
PMSF: Phenylmethylsulfonyl fluoride
